# Reasons for and against presymptomatic genetic testing in frontotemporal dementia: a qualitative study

**DOI:** 10.1007/s00439-025-02795-1

**Published:** 2026-02-16

**Authors:** Charlotte H. Graafland, Harro Seelaar, Jessica L. Panman, John C. van Swieten, Eline M. Bunnik, Laura Donker Kaat

**Affiliations:** 1 Department of Public Health, Program Medical Ethics, Philosophy and History of Medicine, Erasmus University Medical Center, Rotterdam, The Netherlands; 2 Department of Neurology and Alzheimer Center, Erasmus University Medical Center, Rotterdam, The Netherlands; 3 Department of Clinical Genetics, Erasmus University Medical Center, Rotterdam, The Netherlands

## Abstract

The majority of individuals at 50% risk of carrying a pathogenic variant causing frontotemporal dementia (FTD) chooses not to undergo presymptomatic genetic testing. To explore reasons of individuals at risk of genetic FTD for and against genetic testing, we conducted a secondary analysis of semi-structured interviews with 28 Dutch individuals at risk of genetic FTD: 11 carriers, three non-carriers and 14 individuals at 50% risk. We analyzed the data using inductive thematic analysis. Reasons in favor of presymptomatic genetic testing included wanting to know and relieving uncertainty, hoping to test negative, relevance for life planning, reproductive decision-making, explaining future symptoms, and contributing to the common good. Reasons against presymptomatic genetic testing were fearing psychological impact, fearing effects on employment, insurability or mortgage, maintaining hope, and no immediate relevance for life planning, partly due to uncertainty surrounding age of onset. Reasons were similar in all groups, but were weighed differently. Decision-making processes were influenced by (expectations of) psychological burden before and after testing, personal experiences with FTD in the family, and whether genetic status was perceived as actionable. Perceived relevance was partly influenced by age (in relation to age of onset), life circumstances and research participation. When supporting individuals at risk of FTD in decision-making regarding genetic testing, genetic counselors should be aware of and tailor counseling to the individual’s age and lived experience with being at risk, experiences with FTD and related disorders in the family, and factors influencing the perceived relevance of genetic status for reproductive or life decision-making.

**Supplementary Information:**

The online version contains supplementary material available at 10.1007/s00439-025-02795-1.

## Introduction

Frontotemporal dementia (FTD) is characterized by clinical heterogeneity with behavioral symptoms (e.g., disinhibition, emotional blunting), aphasia and/or motor problems (Seelaar et al. 2011). The first symptoms are usually subtle and initially not recognized as consequences of dementia (Bruinsma et al. 2022; Rasmussen et al. 2019; van Vliet et al. 2011), especially as they frequently manifest at a relatively young age before 65 (Harvey et al. 2003). Patients may develop apathy or disinhibition, lose their job or experience changes in (family) relationships, and often lack disease insight (Caceres et al. 2016; Gray et al. 2023; Griffin et al. 2016; O’Keeffe et al. 2007). Overall, patients’ and caregivers’ lives are heavily impacted by FTD, in particular when still raising children or working. There are currently no treatments for FTD that can slow or prevent symptoms.

In 20–30% of cases, FTD is caused by a heterozygous mutation in one of the three major genes: *progranulin* (*GRN*), *microtubule-associated protein tau* (*MAPT*) or *chromosome 9 open reading frame 72* (*C9orf72*) (Greaves and Rohrer 2019). Carriers of a pathogenic variant have an increased risk of developing FTD (and/or amyotrophic lateral sclerosis (ALS), in case of *C9orf72*) at an early age, e.g., 90% of *GRN* mutation carriers will develop FTD by age 70 (Benussi et al. 2015), while penetrance rates in *C9orf72* are variable across families. Children of carriers have a 50% risk of inheriting the mutation. Since over two decades, it is possible to conduct presymptomatic genetic testing to determine an individual’s genetic status (Riedijk et al. 2009). It is standard practice to offer genetic counseling to individuals seeking genetic testing, with the aim to facilitate a well-informed decision about genetic testing and to provide psychological support after disclosure of genetic test results (Crook et al. 2017). Counseling is modeled after the gold standard for counseling for presymptomatic testing in Huntington’s disease (HD), and includes: (1) a pre-test counseling session covering the testing procedure and possible results, the individual’s reasons for and against testing, potential impacts, etc., (2) a recommended reflection period of one month, (3) a disclosure session of the test result, in which implications for clinical management, life choices and family members are discussed and (4) post-disclosure follow-up, often by telephone (MacLeod et al. 2013).

Before genetic testing for FTD was available, a Dutch study suggested that up to 68% of individuals at risk would be willing to undergo presymptomatic genetic testing (Tibben et al. 1997). However, rates of uptake proved much lower once presymptomatic genetic testing had become available, with an initial uptake of 7–17% in genetic FTD (Riedijk et al. 2009), although demand may be increasing in recent years (Amador et al. 2020). Demand for genetic tests in other autosomal dominant neurodegenerative diseases, such as Huntington’s disease, has been similarly discrepant with expressed interest before these tests became available (Mastromauro et al. 1987; Meissen and Berchek 1987; Morrison et al. 2011). Reasons against presymptomatic genetic testing reported in Huntington’s disease, ALS and autosomal dominant Alzheimer’s disease include the lack of effective treatments, the potential negative psychological impacts of positive genetic test results, concerns about effects on insurance or employment, and no perceived relevance for reproductive or life decisions (Anderson et al. 2019; Fanos et al. 2004; Crook et al. 2017), while reasons in favor of testing include hoping to test negative, wanting to know and relieving uncertainty about genetic status, and the usefulness of a genetic result for (reproductive) planning (Crook et al. 2017, Fanos et al. 2004, Scuffham et al. 2014). Individuals at risk of genetic FTD may have similar reasons (not) to pursue genetic testing, but there is little literature on this topic, as previous studies involved only few individuals at risk of genetic FTD (Crook et al. 2017). To our knowledge, only three empirical studies focus specifically on individuals at risk of genetic FTD: one about lived experiences of genetic risk and identity (Dratch et al. 2024), one about reproductive decision-making (Fahy et al. 2025) and one on attitudes towards presymptomatic genetic testing before it was available (Tibben et al. 1997). Since the introduction of presymptomatic genetic testing for FTD, there have been several developments that may have influenced views on genetic testing. Firstly, societal knowledge of genetics and access to internet have improved, so individuals with a family history of FTD can more easily access information about the genetics of FTD and may be more interested in learning genetic information. There may also be generational differences in attitudes towards genetic testing. Secondly, upcoming clinical trials for genetic FTD (Buccellato et al. 2024) may influence at-risk individuals’ decision-making about genetic testing, as only known carriers are eligible, and participants thus need to be aware of their genetic status in order to participate.

Overall, there is a lack of insight into the reasons of individuals at risk of carrying genetic FTD to pursue or decline testing. This study aims to fill this gap by reporting the results of a secondary analysis of interviews with known carriers, known non-carriers and individuals at 50% risk of genetic FTD, which were conducted in a research project focused on the ethics of onset-predictive biomarker tests for FTD (Graafland et al. 2025). The results may help genetic counselors and social workers to gain insights into the reasons that individuals at risk of genetic FTD consider when deciding about presymptomatic genetic testing.

## Methods

### Sampling and recruitment

This study reports the results of an interview study that was conducted in the context of a research project exploring the perspectives of individuals at risk of genetic FTD on new onset-predictive biomarker tests (Graafland et al. 2025). As onset-predictive tests will not be offered to confirmed non-carriers, the original inclusion criteria were: (a) being ≥ 18 years old, (b) carrying or being at 50% risk of carrying a pathogenic variant in a gene known to cause genetic FTD, (c) having no clinical symptoms within the FTD spectrum (behavioral changes, primary progressive aphasia, parkinsonism or motor neuron disease). Participants were recruited from two contexts: (1) L.D.K. recruited individuals who had sought genetic counseling for FTD at the Department of Clinical Genetics at Erasmus MC University Medical Center, (2) H.S. recruited participants for this study from the ongoing FTD-RisC longitudinal cohort study. This study has been running at Erasmus MC, University Medical Centre Rotterdam, since 2009 to study the natural history of genetic FTD by (bi)annually collecting data from known carriers and individuals at 50% risk (Dopper et al. 2014). H.S. and L.D.K. used purposive sampling to select participants from clinical practice and FTD-RisC who were diverse in gender, age and level of education. We aimed to recruit equal numbers of confirmed carriers and individuals at 50% risk.

The interview guide also contained questions about respondents’ reasons for (not) undergoing presymptomatic genetic testing. As we noted the lack of literature on this topic, reasons for and against genetic testing became a secondary focus of the research project. For the current study, we recruited additional confirmed non-carriers to make sure that their perspectives were represented in the data collected from individuals who had undergone genetic testing. When data saturation was reached, defined as no new themes emerging in three subsequent interviews (Guest et al. 2006), we had recruited 11 confirmed carriers, three confirmed non-carriers and 14 individuals at 50% risk.

### Interviews

The interview guide (see Supplement 1) was drafted by the research team (neurologists, clinical geneticists, ethicists and neuropsychologists), and included six questions about respondents’ reasons both for and against genetic testing, regardless of their final decision, to gain insight into the extent to which individuals at 50% consider both sides of the decision.

C.G., a female ethicist and researcher trained in qualitative methods, conducted semi-structured interviews between September 2023 and August 2024 at a location determined by participant preferences, usually in their home or at Erasmus MC. Participants provided written informed consent before the start of the interview. Interviews were audio-recorded, transcribed verbatim using transcription software of Amberscript Global B.V., and manually checked for correctness by C.G.. C.G. made field notes about the most notable findings immediately after each interview.

### Data analysis

Pseudonymized transcripts were analyzed thematically (Braun and Clarke 2006) using NVivo R1 (2020) software. C.G. and E.B. conducted open coding of the first two interviews together, after which they discussed and agreed upon a draft codebook to use for subsequent transcripts. From the beginning of the coding phase, reasons for and against presymptomatic genetic testing were part of the coding process. All transcripts were coded independently by two coders (C.G. in combination with E.B., J.L.P. or one other colleague with experience in qualitative research), and codes were compared and discussed in regular meetings to reach consensus about the final coding. Finally, the content of the codes and their interrelations were analyzed to arrive at the themes reported in this paper. The study design and recruitment are reported following COREQ guidelines (see Supplement 2) (Tong et al. 2007).

## Results

### Participant characteristics

Thirty individuals were invited for research participation, of whom two declined, one because they were busy, and one because they wanted to focus on other things than FTD at this time. Twenty-eight participants were included (12 from the Department of Clinical Genetics, 16 from FTD-RisC). Participants and/or one of their parents carried a hexanucleotide repeat expansion in *C9orf72* (n = 18) or a pathogenetic variant in *GRN* (n = 6), *MAPT* (n = 3) or *TAR DNA-binding protein* (*TARDBP)* (n = 1). Interviews had an average duration of 62.3 min (range 23–92 min). 14 of the participants had undergone genetic testing (11 known carriers, three non-carriers), and 14 participants were at 50% risk, including five who were planning to pursue presymptomatic genetic testing in the near future. Eight of the 14 participants at 50% risk had had genetic counseling, six had not. Known carriers and known non-carriers had received the genetic testing result between three weeks and fifteen years before the interview. The average age of participants was 43.7 years (range 20–66). Table 1 shows an overview of demographic details of participants.

**Table 1:** Demographic details of participants

	Total	Known carriers	Known non-carriers	At 50% risk
Number of participants	28	11	3	14
*Gender*				
Male	12	3	2	7
Female	16	8	1	7
*Age*				
Average age	43.7	45.5	45	42.1
20 to 39 years	10	2	1	7
40 to 59 years	16	8	2	6
> 60 years	2	1	0	1
*Relationship status*				
Single	3	1	1	1
Partner	21	9	2	10
Divorced	4	1	0	3
*Number of children*				
0	10	4	2	4
1 or 2	14	6	0	8
> 3	4	1	1	2
*Genetic group*				
* C9orf72*	18	7	1	10
* GRN*	6	3	1	2
* MAPT*	3	1	1	1
* TARDBP*	1	0	0	1
*Education*				
Low (ISCED^a^ 1–2)	2	0	1	1
Intermediate (ISCED 3–4)	9	4	0	5
High (ISCED 5–6)	11	3	2	6
Higher (ISCED 7)	6	4	0	2

^a^ISCED: International Standard Classification of Education

### Findings

The results were similar in carriers, non-carriers and individuals at 50% risk, unless otherwise stated. They are reported using three themes: reasons in favor of presymptomatic genetic testing, reasons against presymptomatic genetic testing, and decision-making process. Quotes illustrating the results are presented in the text and in Supplement 3 (S3). An overview of reasons in favor of and against genetic testing is listed in Table 2.

**Table 2:** Overview of reasons for and against presymptomatic genetic testing and subthemes

Reasons for presymptomatic genetic testing
Wanting to know and relieve uncertainty
Believing that genetic status is relevant for decision-making - To plan future care - To plan life experiences and work - To support reproductive decision-making - Relevance increases with age
Hoping to test negative - To be able to stop worrying - To relieve hypervigilance, especially in older age - To eliminate the possibility of having passed the gene on to children
Informing children of risk status for reproductive decision-making
Explaining future symptoms
Contributing to common good - To make FTD more well-known in network - To participate in clinical trials

Reasons against presymptomatic genetic testing
Fearing psychological impact of testing positive - Depression or defeatism (for shorter period of time if testing in older age) - Increased hypervigilance - Not wanting to adapt life decisions to carrier status - Change in self-image
Maintaining hope that one is not a carrier
Perceiving no immediate relevance of genetic status - Age of onset remains unpredictable - Reduced penetrance - No effective treatment available for FTD - Can participate in observational research without undergoing presymptomatic genetic testing
Fearing that carrier status affects employment, insurability or mortgage

### Theme 1: Reasons in favor of presymptomatic genetic testing

Participants often mentioned several reasons in favor of presymptomatic genetic testing, in six categories. Firstly, a frequently mentioned – and for some participants, the main – reason was simply wanting to know. Most of the participants who had tested were preoccupied with worrying about their genetic status and preferred the certainty of being a carrier to the uncertainty of being at 50% risk (S3, Q1). Some had already assumed they were carriers when making life choices, and wanted to confirm that their assumptions were correct and their life choices justified.

Secondly, most participants who had genetic testing stated that genetic results have immediate relevance for decision-making in the following domains: (a) planning future care, also to prevent burden on their family (S3, Q2), (b) making reproductive decisions (S3, Q3), (c) planning life experiences and work schedules or retirement. For example, one participant said:

“The advantage of knowing [is that] I can now make decisions, like, ‘What do I want to do in life? Or experience? Or learn?’ [Now,] I don’t think, ‘I will do that after I retire.’ I can make sure that I retire earlier.” (R5, carrier, 40-50 years)

Some participants said that the relevance of genetic status for life planning increased with age, as time until onset would be shorter.

Thirdly, some participants were hoping they would test negative, so they could stop worrying about developing FTD or about having passed on the mutation to their children.

“I just hoped. ‘If I test negative, then I never have to worry about it again. And it would be great for the children, then I could just tell them [the negative result] and that they were spared.’ [Testing] was just a gamble. A stupid gamble.” (R14, carrier, 50-60 years)

Some participants said that they hoped that testing negative would reduce anxiety about their current behaviors being the first symptoms (‘hypervigilance’). Self-monitoring and hypervigilance were expected to increase with age, e.g., when approaching the age of onset of their affected parent or other family members, as the age of onset in family members often influenced their expectations regarding their own age of onset (S3, Q4). Therefore, some participants said that genetic testing would become more appealing in older age, as the burden of increased self-monitoring could be relieved by testing negative.

Fourth, most of the older participants with children wanted to provide their children with information about their genetic risk to support their children’s reproductive decision-making. Sometimes, this was the sole reason for presymptomatic genetic testing, or it would be in the future, when the participant’s children reach the reproductive age (S3, Q5).

The fifth reason that some participants mentioned in favor of presymptomatic genetic testing was that positive results could be used to explain future symptoms. Many participants experienced that it took years to find out that their parent’s first subtle behavioral changes were due to dementia. Some thought that knowing about carrier status may help family members to interpret early behavioral changes correctly rather than blaming the person with FTD for inappropriate behavior, thus supporting early diagnosis and timely intervention. One participant said:

“What if you don’t get tested, but things do start happening that (…) makes your family think, ‘Why is he acting weird?’ Without knowing the cause. (…) And [when you have tested], it is still frustrating [to have symptoms], but you do know where they are coming from.” (R17, carrier, 40-50)

Finally, some participants wanted to contribute to the common good through genetic testing, either by making FTD more well-known in their network, or by becoming eligible for participation in clinical trials of investigational treatments for FTD (S3, Q6). Regarding the latter, a few participants thought that genetic testing would help them “get on the list” of researchers recruiting for clinical trials, whereas they might not be approached for trial participation if they were unaware of their genetic status (S3, Q7).

### Theme 2: Reasons against presymptomatic genetic testing

Participants, both those who tested and those who did not, mentioned four reasons not to undergo presymptomatic genetic testing. Firstly, many participants worried about the psychological impact of receiving positive results. Some participants were sure they would not be able to cope with that news, for example, one participant said:

“I just know that [a positive genetic result] won’t sit well with me. Just mentally, what is the purpose of knowing you are going to end up that way [with dementia]? I think [I would be] bordering on depression.” (R16, 50%, 20-30 years)

Others were unsure how they would react. Participants considered four potential negative psychological consequences of testing positive: a) intense sadness or depression (S3, Q8), b) increased hypervigilance (S3, Q9), c) being forced to make life changes, while preferring to live life as one did with 50% risk (S3, Q10), d) negative effect on self-image, especially regarding one’s mental capacities. Several participants described it as feeling like a “ticking time bomb” with an unknown amount of time left on the clock (S3, Q11). Some respondents believed that age at disclosure of genetic testing results would influence psychological impacts: in older age, there are fewer years left to worry about onset, compared to testing positive at a younger age (S3, Q12).

Secondly, some participants wanted to maintain hope that they were not a carrier:

“If you know and you have it, what does it do to your life? Now I still [have] a bit of hope, and where there’s hope, there’s life. (…) If you have it [the gene], you might throw away (…) years where you are thinking about it in a different way. You throw away a certain kind of joy.” (R7, 50% risk, 50-60 years)

Some participants characterized presymptomatic genetic testing as an irreversible decision: once you know your genetic status, you cannot “unknow” it. Some participants believed that living with 50% risk, though burdensome, was less burdensome than knowing that one is a carrier. At the same time, some participants at 50% risk though that this balance might shift as hypervigilance increased when they would get older and approach the age of onset in their family member(s), which some took as an indication of the age at which they might develop symptoms. A few other participants did not perceive their 50% risk as burdensome in daily life (S3, Q13). The wish to maintain hope of not being a carrier was especially present in younger participants, as they stated that they could always undergo presymptomatic genetic testing later, when approaching the age at which family members developed symptoms.

Thirdly, many participants who had chosen not to undergo presymptomatic genetic testing stated that their genetic status did not have immediate relevance for current decision-making. Some participants partly attributed this perceived lack of relevance to the unpredictability of age of onset. Genetic testing might indicate *that* one is going to develop FTD, but not *when* (S3, Q14), while the age of onset could be important for planning, e.g., whether to save for retirement or not. In addition, a few participants pointed out that 10% or more of carriers do not develop FTD before they die of other causes. For these individuals, any life decisions based on genetic status would have been for nothing.

“[If you test positive], you know that you will become ill with 90 percent certainty. So it could also be that you adapt your whole life, and yet you are in the 10 percent that do not become ill. (…) And you also do not know when it starts.” (R25, 50% risk, 40-50 years)

Additional reasons that genetic information was perceived as irrelevant by participants included the current lack of effective treatment options for FTD (S3, Q15), and opportunities to participate in observational research studies without knowing one’s carrier status; being monitored and notified when developing symptoms removed the need to know one’s genetic status (S3, Q16). Another participant mentioned how she no longer felt a need to undergo genetic testing after her sibling had tested negative. Her sister would be able to take care of her if she became symptomatic, so she no longer needed her genetic status to make arrangements for long-term care (S3, Q17).

Finally, some participants were afraid that testing positive might affect their own or their family members’ chances of getting a mortgage, finding or maintaining employment or purchasing life or disability insurance (S3, Q18).

### Theme 3: Decision-making process

Each participant went through a personal decision-making process after first learning about their 50% risk and the possibility of presymptomatic genetic testing. Some participants said that they made an almost immediate initial decision either to test or not to test at that time, sometimes based on their emotional reaction to the news. For example, one participant said:

“Then my father tested positive. Yes, then I automatically also [wanted to] get tested, and unfortunately, I am also positive.” (R1, carrier, 30-40 years)

Other participants said that they first took a period of time (e.g. a few months) to process that they were at 50% risk, and then started considering whether they wanted to undergo presymptomatic genetic testing. We observed that some weighed reasons for and against testing, while others saw only reasons for either testing or not testing. Thirdly, some participants said that they did not actively consider their risk or the possibility to undergo presymptomatic genetic testing at first, for example because participants were preoccupied with the care for their affected parent (S3, Q19).

Participants who initially did not pursue genetic testing sometimes said that considerations around genetic testing changed over time. Some of them believed that they might pursue testing in the future, but did not want to receive their genetic result at this time, because of several reasons. For example, they first wanted to arrange disability insurance or a mortgage or were waiting for other issues to blow over so they would have mental space to process genetic test results (S3, Q20). In one case, the participant’s parent had a 50% risk of carrying the mutation but had not wanted to undergo genetic testing, so the participant waited until FTD manifested in their parent before undergoing genetic testing herself (S3, Q21). Other participants who had initially decided not to undergo genetic testing mentioned that they sometimes reconsidered this over time, for example after the death of an affected parent or other experiences with FTD in the family, when they believed that they may be developing symptoms, or when they perceived new utility of learning their genetic status, e.g., for reproductive planning of themselves or their children (S3, Q22). In some cases, this period of reconsideration led them to pursue genetic testing after all. For example, a participant attended the disclosure session of his brother, who tested negative, leading him to pursue testing as well (S3, Q23). Overall, we observed that considerations surrounding genetic testing could change over time, with preliminary decisions made at several time points, that could later be reconsidered.

We also observed that decision-making about presymptomatic genetic testing was influenced by two factors. Firstly, most participants engaged in discussions about testing with other people, albeit to differing extents. Important interlocutors were partners, siblings, children and parents, and in some cases cousins and friends. One participant spontaneously mentioned that she was influenced by social media posts describing negative psychological impacts from receiving positive results.

“On Instagram I searched for the tag of the gene. Then I found a few people, and I just started a conversation with them, also a few young girls who knew [their status]. And all five, they said, ‘I wish that I hadn’t [tested], that I didn’t know.’” (R12, 50% risk, 30-40 years)

How strongly other people influenced the participant’s decision varied. Some participants involved their partner to such an extent that they spoke about the decision in terms of “we” (S3, Q5, Q24). In other cases, the partner simply expressed support for whatever decision the participant made. Some participants made the decision mostly from a sense of duty towards their children: one participant recounted how her children asked her to reconsider testing, so that the results might inform their reproductive decision-making (S3, Q25).

For participants who had genetic counseling, we observed that this was a second factor influencing decision-making, although the influence of counseling differed per person. Some participants thought counseling had facilitated their decision-making process or had confirmed their thought process, by providing more arguments to support it or offering additional information, e.g., about the experiences of other individuals at risk (S3, Q26). Others had already gathered information about genetic testing from family members or the internet and had made preliminary decisions about testing before counseling. In these cases, genetic counseling did not provide new information or change the final decision (S3, Q24).

## Discussion

This is the first empirical study focused on reasons for and against presymptomatic genetic testing in FTD since genetic testing is available. Previous literature, predominantly reporting on survey studies, lists similar main reasons for and against testing in other genetic neurodegenerative disorders, including HD (Amador et al. 2020; Anderson et al. 2019; Binedell et al. 1998a, b; Etchegary 2006; Klitzman et al. 2007; Rivera-Navarro et al. 2015; Scuffham and MacMillan 2014; Wedderburn et al. 2013; Williams et al. 2010), autosomal dominant Alzheimer’s disease (Tibben et al. 1997), genetic forms of ALS (Benatar et al. 2016; Fanos et al. 2004; Fanos et al. 2011) and ALS and/or FTD (Amador et al. 2020; Crook et al. 2017, 2021; Surampalli et al. 2015; Tibben et al. 1997). This qualitative study adds to the existing literature by confirming that reasons like wanting to know, the (ir)relevance of genetic status for decision-making and fear of psychological impacts also play a role in the decision-making of individuals at risk of genetic FTD. It also reports some subthemes that have not been described before, such as the role of reduced penetrance and ongoing participation in observational research in reducing the relevance of genetic status for reproductive or life decision-making, and higher age as increasing the relevance of testing. Some of these results have implications for the clinical practice of genetic counseling and care for individuals at risk of genetic FTD.

Firstly, the results of this study point to the significant psychological impact of being at 50% risk. One prominent reason in favor of genetic testing was relieving uncertainty about genetic status, as participants who had tested thought uncertainty would be more burdensome than the certainty of a positive genetic result. In addition, participants who had not tested tended to experience hypervigilance, which they expected might increase with age. There is very little research on the lived experience of being at 50% risk of FTD and on what resources these individuals need to cope well with their risk, such as peer support or professional psychological support. As the burden of being at risk may increase over time, healthcare professionals or researchers who regularly interact with individuals at 50% risk of FTD may inquire after changing experiences of risk over time, and if needed, refer them to resources for support.

An interesting difference between individuals who had tested and those who had not was that the tested individuals often perceived their genetic status as relevant for life planning, whereas individuals at 50% risk generally did not, partly due to reduced penetrance and uncertainty regarding the age of onset. Overall, it may be helpful to discuss the influence of age and uncertainty regarding age of onset on the perceived relevance of genetic status in pre-test counseling. In the future, uncertainty about the age of onset may be reduced by the development of new predictive biomarker tests, that could predict the onset of symptoms a few years in advance. As reported by Graafland et al. (2025), some individuals at 50% risk would be willing to learn their genetic status if the onset-predictive test indicated that onset would occur within a few years’ time. Onset prediction would be more actionable, allowing for preparations regarding care, end of life wishes, and professional and private life. Thus, the considerations of participants at 50% risk regarding genetic testing may change in the future with new test and treatment developments, and should be re-investigated as the landscape of FTD biomarker testing changes.

The perceived relevance of genetic testing was influenced by the presence of opportunities to participate in research studies, with observational research and clinical trials having different effects. On the one hand, participation in longitudinal, observational research could reduce the perceived relevance of genetic information for participants at 50% risk. The FTD-RisC study and the European Genetic Frontotemporal dementia Initiative (GENFI) consortium, for example, do not require participants to learn their genetic status before participation. Participants undergo extensive cognitive testing, MRI, blood draws and sometimes lumbar punctures in (bi)annual study visits, and will be alerted if they are developing symptoms of FTD. These regular tests were perceived as reassuring check-ups by some interview participants and made learning their genetic status less relevant. On the other hand, some participants were led to *pursue* genetic testing by a desire to participate in clinical trials; they wanted to “get on the list” for clinical trials that require participants to know their genetic status. Our findings indicating a strong motivation to participate in research and clinical trials on FTD align with those of an interview study among American individuals at risk of FTD (Dratch et al. 2024). This motivation may be driven by the current lack of effective treatments for FTD. Research may be seen as the only way to move forward and expand options for clinical management of FTD (Crook et al. 2021). This reason to undergo genetic testing may become more prevalent as more clinical trials for different FTD variants become available in the future, but it also raises ethical questions. Individuals at 50% risk may feel pressured into undergoing pre-symptomatic genetic testing as an – only – way to gain access to investigational treatments through research participation. They might have inflated expectations of medical benefits from participation in clinical research, while they may (also) have good reasons *not* to undergo testing. Clinical geneticists should carefully explain the nature of clinical research (including, e.g., the possibility of being randomized to placebo control groups), the risks and burdens involved, and the limited chance at benefit that research participation may have for individual participants.

Finally, decision-making on genetic testing also seems to be influenced by person-specific factors, including previous experiences with FTD, genetic testing in the family, the diagnostic journey of the parent, and even information obtained from social media. Firstly, the type of FTD and symptoms in the family influenced participants’ perceptions of the nature, age of onset, clinical presentation and quality of life of FTD, which may all influence decision-making about genetic testing. For example, if the participant viewed their family member’s disease trajectory or death as horrible and undignified, they tended to see genetic testing as a way to prepare and avoid meeting the same fate. Secondly, previous experiences with genetic testing in the family sometimes influenced decision-making, either in favor of testing (if results had been negative) or against (if, for instance, family members had trouble coping with positive results). Thirdly, if the participant’s parent had had an extended diagnostic journey, participants were more prone to feel that knowing about being a carrier would help their family to recognize symptoms at an early stage. A similar perceived advantage of knowing about carrier status was found in HD (Williams et al. 2010). However, in reality, these individuals and their families are already aware of their genetic risk, so a diagnostic odyssey is unlikely when symptoms present, even in the absence of a genetic test result. Genetic counselors should make individuals at risk of FTD aware of the differences between them and their parents in this regard. A fourth factor that may influence individuals’ understanding of genetic testing is social media, which has not been mentioned in previous literature. In this study, one participant mentioned how social media had made her more aware of potential negative impacts of testing. The influence of social media may increase in the future, as generations who have grown up with social media are now becoming old enough to decide about genetic testing. There is a potential for misinformation to spread via social media that counselors should be aware of. More research is needed to understand how access to the internet and social media may influence decision-making about genetic testing in younger generations. In genetic counseling, it may be helpful to ask what information individuals have already collected from family members or the internet, in order to correct any misunderstandings.

### Limitations

The results of this study may have been influenced by several limitations. Firstly, this study was focused on onset-predictive biomarker testing (Graafland et al. 2025), with reasons for and against presymptomatic genetic testing as a second focus. Although the interview guide included six questions on decision-making regarding presymptomatic genetic testing, the topic may have been more extensively explored had it been the primary aim of the study.

Secondly, the study was conducted only in the Netherlands and included mainly highly educated respondents. Culture, education and societal context might influence how individuals make decisions regarding presymptomatic genetic testing, e.g., access to education and health care may determine how well individuals understand the concept of genetics or whether they can access genetic testing or counseling. For example, all respondents were recruited in specialist care settings. In the Netherlands, it is common for potential carriers of pathogenic FTD variants to be referred to specialist care settings. Access to specialist care – or lack thereof – may influence potential carriers’ decision-making about presymptomatic genetic testing, and consequently, our results cannot be extrapolated to other countries with different healthcare systems. Also, the results may not apply to individuals at risk who have not sought genetic counseling. Furthermore, we did not gather any data on religion or level of religiosity, or on employment status. Future studies could explore whether and how reasons for and against genetic testing differ with country of residence, religious group or employment status.

Thirdly, the number of participants was too small to study differences between groups carrying different variants. For example, *C9orf72* hexanucleotide repeat expansions also confer a risk of developing ALS instead of or in addition to FTD and generally have lower penetrance; these differences may influence decision-making. Future studies could look into potential differences in genetic testing decisions between genetic groups.

Furthermore, only three study participants were confirmed non-carriers, which means their views on genetic testing may be underrepresented in the sample. Still, it seems likely that the considerations for and against genetic testing are similar in non-carriers and carriers, since prior to testing, both groups are in the same state of uncertainty regarding carrier status prior to testing. Also, we did not find large differences in considerations regarding genetic testing between carriers, individuals at 50% risk and non-carriers.

Finally, there was a wide range in the length of time participants had known about their 50% risk or genetic status before the interview, ranging from three weeks to fifteen years. The longer the period of time, the higher the likelihood of recall bias and post-hoc rationalization of reasons for and against presymptomatic genetic testing. In some cases, respondents’ remembered reasons for and against genetic testing may not accurately reflect their actual considerations at the time of testing.

## Conclusions

This study is the first qualitative study on the reasons for and against pre-symptomatic genetic testing for FTD after genetic testing became widely available in the Netherlands. Reasons in favor of genetic testing include wanting to know and relieve uncertainty, believing that genetic status is relevant for decision-making, hoping to test negative, informing children of risk for reproductive decision-making, explaining future symptoms and contributing to the common good. Reasons against genetic testing are fearing psychological impact of testing positive, maintaining hope that one is not a carrier, perceiving no immediate relevance of genetic status, and fearing that carrier status affects employment, insurability or mortgage. Participants who underwent genetic testing considered the same reasons as participants who did not, but weighed them differently. Uncertainty about age of onset and access to research participation seem to influence the perceived relevance of genetic status for decision-making. In addition, the individual’s previous experiences with FTD, genetic testing in their family, the diagnostic journey of their parent, and information obtained from social media may influence their decision-making regarding presymptomatic genetic testing in FTD. These insights may support genetic counselors in addressing challenges specific to FTD in genetic counseling and provide tailored care to individuals at risk of genetic FTD.

## Supplementary Information

Below is the link to the electronic supplementary material.

Supplementary file1 (DOCX 25 KB)

Supplementary file2 (PDF 485 KB)

Supplementary file3 (DOCX 45 KB)

## Data Availability

The datasets generated and/or analyzed during the current study are not publicly available to protect the privacy of participants with rare genetic risk for FTD. The datasets are available from the corresponding author on reasonable request.

## Abbreviations

ALS: Amyotrophic lateral sclerosis
C9orf72: Chromosome 9 open reading frame 72
Erasmus MC: Erasmus MC University Medical Center
FTD: Frontotemporal dementia
GENFI: The genetic frontotemporal dementia initiative
GRN: Progranulin
HD: Huntington’s disease
ISCED: International standard classification of education
MAPT: Microtubule-associated protein tau
S3: Supplement 3
TARDBP: TAR DNA-binding protein
