# Supplementary material for: Reasons for and against presymptomatic genetic testing in frontotemporal dementia: a qualitative study
Source: Hum Genet. 2026 Feb 16;145(1):21. doi: 10.1007/s00439-025-02795-1 (PMC12909385; doi:10.1007/s00439-025-02795-1)
Supplement: Supplementary file 3 — Supplementary file3 (DOCX 45 KB) [file 439_2025_2795_MOESM3_ESM.docx]

Supplement 3: Quotes illustrating interview results

|  | Participant, genetic status, age | Quote |
| --- | --- | --- |
| Q1 | R23, carrier, 30-40 | I am someone who wants to know everything. I love certainty, so I did the test, because I don’t want to have the daily feeling, ‘Do I have it?’ (…) I can’t handle that. I can handle it better if I know whether I have it or not, because then I can put it aside. |
| Q2 | R2, carrier, 40-50 | If I get something [symptoms], then I just don’t want to be a burden on the people around me. Because then I would like to (…) arrange something, [so] that I can just be taken care of somewhere else. (…) That’s one reason I chose to test. |
| Q3 | R1, carrier, 30-40 | For us, whether we wanted children, yes or no, was also dependent on [the genetic result] (…) From the moment that I heard that it could be hereditary, I decided pretty quickly that, if I am positive, I do not want children. |
| Q4 | R3, 50% risk, 30-40 | The older you get, the greater the chance [that you develop symptoms], of course. I think if you start approaching that age at which all your family members got it [FTD], you will more quickly think, ‘Hey, is this it?’ Or I can even imagine, if that uncertainty becomes too great at a certain point, that you start reconsidering whether you want to test or not. |
| Q5 | R6, 50% risk, 50-60 | The only reason that we would want to know is if there is something with the children. Well, and when does that become relevant? Only when they are thinking about having children themselves. |
| Q6 | R12, 50% risk, 30-40 | If you get tested, and you have it, then you can participate in a study and then you can really help by actively taking medication. (…) [I would] say for the common good: ‘Yes, [I am] a good candidate [for participation in a clinical trial]. Let’s do it [genetic testing].’ |
| Q7 | R8, carrier, 40-50 | From now on, [now that I know I am a carrier,] I can participate in research. My sister joked, ‘Do you think they have a list and they put you at the top: call this one first?’ I said: ‘People who do not get tested and do not participate in research, they are not on the list at all. (…) From now on, I will be at the front of the line if I get the chance.’ |
| Q8 | R5, carrier, 40-50 | There is the disadvantage that if you have the gene, of course, that makes you quite sad and you’re crushed by it, and it may leave a mark for the rest of your life. So I thought I should not underestimate that. |
| Q9 | R6, 50% risk, 50-60 | If you have it, every time that you forget a name, you think, ‘Jeez, is this it [the first symptom]?’ |
| Q10 | R6, 50% risk, 50-60 | If I would know, I would perhaps start to live my life in a different way, a way that I do not feel like right now. |
| Q11 | R12, 50% risk, 30-40 | You are a sort of a ticking time bomb I think, and that feeling is already there when you don’t test. And if you test positive, [that feeling] worsens. And only if you get tested and you don’t have it, does it go away. |
| Q12 | R27, non-carrier, 40-50 | When it’s bad news, then (…) you have a long time to go. Especially since the disease starts between 50 and 60. Yes, then you have to live towards it for a long time. (…) Age makes a difference: whether you are five years from [age of onset] or you are almost 25 years away from it. |
| Q13 | R26, non-carrier, 50-60 | I quite quickly thought, ‘I am not very worried about it right now. How will I react if I know? What would life look like then?’ (…) And in that moment, I could pretty easily put it aside and just put it in God’s hands, like: yes, it can be so and it cannot be so. |
| Q14 | R21, carrier, 60-70 | So to another person I [would] now say: ‘Don’t do it, because you substitute one uncertainty [about genetic status] with the other [about age of onset].’ |
| Q15 | R12, 50% risk, 30-40 | You can’t do anything about it. Look, if you carry a very nasty gene, like, for example, the breast cancer gene, that is also very intense, because then you start to consider things like an amputation or a hysterectomy. And [with FTD], you can’t. Well, then you know, and then what? There is no preventive measure that you can consider then. |
| Q16 | R22, 50% risk, 30-40 | Actually, we just decided: we probably have it. Period. And we are in a study where we are followed on an anonymous basis, but if there are reasons to contact you, they will. (…) I see it like this: you actually get an annual checkup without being kept up-to-date, which is nice in a way. |
| Q17 | R25, 50% risk, 40-50 | The relief [that my sister tested negative] was enormous, because it meant that [my sister] would remain healthy. (…) It also gives a lot of space, also for me, not to have to know. If she would have been a carrier, then I would also test, because then I would want to know if I could be there for her. |
| Q18 | R9, carrier, 40-50 | It does put a mark on you, even though you’re not really sick. I find that difficult. For me it doesn’t have much effect, but it would be an issue if you couldn’t get a mortgage, because you had a mark of carrying a gene that could perhaps make you sick. |
| Q19 | R28, non-carrier, 30-40 | Interviewer: What was the reason that you did not get tested in the beginning [three years before]? Because you kind of assumed that you had it, but…  R28: Yes, first I was busy with my father [who needed care]. [I still am] now, of course, but, you know, my mother also needed support. |
| Q20 | R19, 50% risk, 30-40 | And then I have to search for a good moment, because I want to run away for at least a week with my husband when I have the result, especially if it is bad news. [I want to] have a moment to get it together. |
| Q21 | R8, carrier, 40-50 | I actually always wanted to get tested, but my father didn’t want to know [his genetic status]. So yeah, if I would have gotten tested then, I would have also been deciding for him. |
| Q22 | R3, 50% risk, 30-40 | There was a moment at the start of this year that I suddenly hesitated, like, ‘Maybe I should just get tested.’ Also because I learned more about this trajectory [preimplantation genetic testing] for the first time. I discovered what an enormous amount of time and effort it takes, also what you do to your own body if you start to inject all these hormones. It makes you think, ‘Jeez, if we could prevent all that [by choosing presymptomatic genetic testing and testing negative], it might be nicer.’ |
| Q23 | R27, non-carrier, 40-50 | [My brother] decided to test. I thought that was very admirable. But then (…) I came with him for the result and then I also talked with [the clinical geneticist] and thought about it in-depth. And I am also, well, [40-50] years old, so you start approaching 50. So then I decided: now we are also going to do it [testing]. |
| Q24 | R25, 50% risk, 40-50 | We have talked to a social worker once, around the time of that [testing] process, to investigate that question [whether you want to test], but actually [my partner] and I had already decided that, so. |
| Q25 | R26, non-carrier, 50-60 | At first I told [my son], ‘I actually decided that I was not going to test.’ (…) Then we had the talk [with the genetic counselor] and then later I thought, ‘If [my children] are really this preoccupied with it, (…) then I would be very egoistic if I decided for myself that I didn’t need to know.’ |
| Q26 | R10, carrier, 40-50 | What I also thought was very good: the doctor also told us, ‘This is what we see with other patients,’ or, ‘Other patients handle it this way.’ |
